# Application of Bayesian Active Learning to the Estimation of Auditory Filter Shapes Using the Notched-Noise Method

**DOI:** 10.1177/2331216520952992

**Published:** 2020-10-19

**Authors:** Josef Schlittenlacher, Richard E. Turner, Brian C. J. Moore

**Affiliations:** 1 Department of Experimental Psychology, University of Cambridge; 2 Department of Engineering, University of Cambridge

**Keywords:** Bayesian active learning, hearing test, auditory filter, notched noise

## Abstract

Time-efficient hearing tests are important in both clinical practice and research studies. This particularly applies to notched-noise tests, which are rarely done in clinical practice because of the time required. Auditory-filter shapes derived from notched-noise data may be useful for diagnosis of the cause of hearing loss and for fitting of hearing aids, especially if measured over a wide range of center frequencies. To reduce the testing time, we applied Bayesian active learning (BAL) to the notched-noise test, picking the most informative stimulus parameters for each trial based on nine Gaussian Processes. A total of 11 hearing-impaired subjects were tested. In 20 to 30 min, the test provided estimates of signal threshold as a continuous function of frequency from 500 to 4000 Hz for nine notch widths and for notches placed both symmetrically and asymmetrically around the signal frequency. The thresholds were found to be consistent with those obtained using a 2-up/1-down forced-choice procedure at a single center frequency. In particular, differences in threshold between the methods did not vary with notch width. An independent second run of the BAL test for one notch width showed that it is reliable. The data derived from the BAL test were used to estimate auditory-filter width and asymmetry and detection efficiency for center frequencies from 500 to 4000 Hz. The results agreed with expectations for cochlear hearing losses that were derived from the audiogram and a hearing model.

The notched-noise method has been widely used in research studies to estimate the shapes of auditory filters (Glasberg & Moore, 1990; Irino & Patterson, 2001; Patterson, 1974; 1976; Patterson et al., 1982, 1995). With this method, the threshold for detecting a sinusoidal signal in a noise with a spectral notch is measured as a function of notch width and the position of the notch relative to the signal frequency. The variation of signal threshold with notch width and asymmetry is used to estimate the shape of the underlying auditory filter. It is thought that the sharpness of the auditory filters is largely determined by the operation of the outer hair cells in the cochlea (Moore et al., 1999). Hence the measurement of auditory-filter shape may be useful for diagnosing the underlying cause of hearing loss (Moore & Glasberg, 2004). Also, if estimates of auditory-filter shape are obtained over a wide frequency range, the results may be useful for the fitting of hearing aids; this is discussed later in this article.

An obstacle to the estimation of auditory-filter shapes in clinical practice is the time taken to obtain the estimates. Using traditional methods, which typically involve the use of nine or more notch widths and estimating each threshold two or three times using an adaptive forced-choice method (Patterson et al., 1982), it takes about 2 hr to estimate the auditory-filter shape at a single center frequency. In what follows, we review methods that have been used, or might be used, to reduce the time taken, either specifically for notched-noise measurements or for threshold estimation in general, focusing especially on Bayesian active learning (BAL). We then describe the application of BAL to the efficient estimation of signal thresholds in notched noise for a wide range of signal frequencies and notch widths.

Stone et al. (1992) and Leeuw and Dreschler (1994) tried to find a reduced set of notch widths that allowed determination of the width and asymmetry of the auditory filter with only a small reduction in accuracy. Stone et al. proposed using five notch widths with two up-down forced-choice threshold runs (Levitt, 1971) for each notch width. This would require about 30 min to estimate the auditory-filter shape at a single center frequency and it comes at the cost of some loss in accuracy relative to the use of a “full” set of notch widths.

Békésy (1947) circumvented the limitation of testing only one frequency at a time for the audiogram by slowly sweeping the signal frequency over time and decreasing the level when the subject indicated that the tone was heard and increasing it otherwise. A similar technique with a variable masker level has been used for measuring psychophysical tuning curves (Sęk et al., 2005), which represent the level of a narrowband masker required to mask a fixed sinusoidal signal as a function of masker frequency. In principle, the method of Békésy could be adapted to the estimation of auditory-filter shapes at different center frequencies, for example, by sweeping the signal frequency and notch center frequency together. Although this procedure is time efficient and samples at informative points around the threshold, it is problematic because subjects may be slow to respond when they stop/start hearing the signal, there may be lapses of attention that affect the measurements even after attention is restored, and the subject may “loose what to listen for,” since only near-threshold stimuli are presented.

Other methods have been developed with the goal of improving time efficiency for a single threshold estimate without losing accuracy compared with forced-choice up-down procedures. The single-interval adjustment matrix procedure (Kaernbach, 1990) does this by considering the receiver operating characteristic, so that a Yes/No procedure can be used but the response criterion is accounted for. This procedure required about a third of the number of presentations as for a two-interval forced-choice method to obtain equal accuracy. However, even with this method, the time required would be too long to allow the estimation of auditory-filter shapes over a range of center frequencies in clinical practice.

An early Bayesian procedure, QUEST (Watson & Pelli, 1983), estimated the detection threshold given the data obtained already. It did this after each trial. The level used in the next trial was the current estimate of threshold. This led to more rapid threshold estimates. Later time-efficient methods placed an emphasis on modeling the unknown response distribution in more detail, for example, estimating the threshold and the slope of a psychometric function (Brand & Kollmeier, 2002).

To our knowledge, the first BAL method in psychophysics that used Bayesian principles for both modeling the response and choosing the parameters for the next trial was introduced by Cobo-Lewis (1997). His method was designed to classify a subject into one of nine audiometric groups, for example, “normal hearing” or “mild to severe sloping loss.” The stimulus for the next trial was chosen to maximize the mutual information between the current estimate and that after obtaining one more response. To do this, the posterior probabilities for all candidates who were considered for the next trial were calculated and the one with the least expected entropy (Shannon, 1948) was chosen. Cobo-Lewis validated the method with numerical simulations.

Kontsevich and Tyler (1999) described a BAL method for estimating the threshold and the slope of a psychometric function, and, like Cobo-Lewis, maximized mutual information when choosing the stimulus for the next trial. They evaluated the procedure with simulations and with real subjects. At that time, computational limits restricted BAL methods to one independent variable only, which was sound pressure level.

Houlsby et al. (2011) presented general BAL methods for classification and preference tasks that used Gaussian Processes (GPs, Rasmussen & Williams, 2006) for modeling a subject’s response probabilistically. GPs can be multidimensional, that is, model several independent variables, and they can incorporate prior beliefs about the mean, the smoothness of the boundaries between response classes and the covariance between data points. The latter allows the experimenter to determine how the threshold changes along a given dimension, for example, whether the detection probability increases with increasing value of the variable (e.g., sound pressure level in many auditory experiments), whether the detection probability changes smoothly when changing the variable by a small amount (e.g., frequency in an audiogram), and whether or not there are interactions between the dimensions. Houlsby et al. (2011) also presented a formula for calculating mutual information without the costly computation of the expected posterior entropy. This was done by exploiting the commutativity of mutual information. The mutual information between the outcome and the model parameters does not require computation of the posterior entropy across the whole space for each candidate data point and outcome (*H(X|Y)*); evaluating the conditional entropy for each data point given the current GP (*H(Y|X)*) is considerably faster.

This approach worked well for determining the similarity between images (Houlsby et al*.,* 2013) and has also been used in auditory applications. For example, GPs have been used to search for the optimal setting of a hearing aid (Jensen et al., 2019; Nielsen et al., 2014), and for determining audiograms (Cox & de Vries, 2015; Schlittenlacher et al., 2018a; Song et al., 2015), equal-loudness contours (Schlittenlacher & Moore, 2020), and psychometric functions (Song et al., 2017). Other BAL approaches, often using parametric models but also maximizing mutual information or something similar, have been used to determine equal-loudness contours (Shen et al., 2018) or the edge frequency of a dead region (Schlittenlacher et al., 2018b).

Most important for the present work, Shen and Richards (2013) and Shen et al. (2014, 2019) determined auditory filters using a parametric BAL approach. Their methods were aimed at estimating the shape of the auditory filter at a single center frequency, and the procedure required about 10 min to determine the width of the auditory filter, and about 15 min to determine both its width and asymmetry.

All of the methods reviewed above would be too time-consuming for use in clinical practice to estimate auditory-filter shapes over a wide range of center frequencies. In this study, we present and evaluate a BAL method that estimates the detection threshold for a signal in notched noise as a continuous function of signal frequency from 500 to 4000 Hz for nine notch widths, with the notches placed both symmetrically and asymmetrically around the signal frequency. We applied nine GPs concurrently, one for each notch width. We theoretically evaluated the information content of different tasks to choose the most efficient task to be used in the BAL method, which was a yes–no task. The BAL method proved to be time-efficient, yielding the desired signal thresholds with good accuracy within 20 to 30 min. Comparisons with a second run performed with a single notch width showed that the outcome is reliable and comparisons with a two-interval two-alternative forced-choice (2I-2AFC) procedure at one center frequency demonstrated its validity.

The data derived using the BAL method were analyzed using a simple model for the auditory filter that had only two parameters, defining the lower slope and upper slope. This allowed the parameters to be estimated accurately in a short time while still characterizing the main features of the filter. In addition, the fitting process included a parameter, *K*, characterizing the combined effects of the subject’s detection efficiency and response criterion.

## Method

In this study, as in most previous studies using notched noise, the noise was composed of two bands, one centered above and one centered below the signal frequency. Hence the stimuli were defined by eight variables: the level and two cut-off frequencies of each noise band and the level and frequency of the sinusoidal signal. Some of the variables need to be fixed to make the duration of the experiment reasonably short, and these were chosen to follow the conventions of previous studies using the notched-noise paradigm. We fixed the signal level (*L_s_*) at 15 dB SL and the bandwidths of the two masking noises at 0.4 times the signal frequency (*f_s_*) in Hz. The two masker bands had the same level (*L_m_*), and a single variable was used to represent the notch condition, with nine instances. The three independent variables were thus *f_s_*, *L_m,_* and notch condition.

A BAL method can either be parametric or threshold-based. Parametric methods have the advantage that they directly maximize the information with regard to the parameters of interest and thus are potentially faster. Threshold-based methods have the advantage that the model parameters can be chosen after the test, that is, more than one model could be fitted. Furthermore, threshold-based methods can be faster to compute when the model is complex or when it has many parameters. We chose to estimate the detection thresholds for tones in noise because the computation of auditory-filter shapes is computationally expensive and this was done after the test rather than between trials. If one wanted to estimate filter shapes directly, one could compute them for a grid of independent variables in advance, as was done by Shen and Richards (2013) and Schlittenlacher et al. (2018b).

This section explains the basics of active learning and GPs and considers what task design is most informative for the present test, before going into the details of the experiments and the procedures for the BAL test and a forced-choice method that was used for comparison.

### BAL Using GPs

For each notch condition, the masker level at threshold needed to be estimated as a function of signal frequency, *f_s_*. A GP was calculated for each notch condition to yield a probabilistic estimate (a Gaussian distribution with a mean and variance) of signal detectability for each point in the two-dimensional frequency-level (*f_s_*-*L_m_*) space:

(1) $$f\left(x_{*},x,y\right)=GP(m\left(x_{*},x,y\right),k\left(x_{*},x\right))$$

with $x_{*}$ a point in frequency-level space, *f* the GP function at $x_{*}$ given already obtained responses ***y*** at frequencies and levels ***x***, *m* the mean and *k* the kernel, which determines the covariance between pairs of data points. We chose a mean of the GP function based on the data already obtained, that is, a scalar mean that was constant for all ***x*** and that was obtained by maximizing the marginal likelihood of ***y*** given **x** and hyperparameters ***θ***, p(***y***|***x***,***θ***), with regard to this single hyperparameter for the mean (for details, see Rasmussen & Williams, 2006, Chapter 5.2). This was done by an iterative optimization procedure, which always started at an initial value of 0 for the mean. The covariance was linear in level, which represents the fact that detectability decreases with increasing noise level, and a squared-exponential kernel in frequency with a length scale of 0.5 octaves was used, which represents the fact that the threshold varies smoothly with frequency.

Equation 1 gives the GP function in latent variable space, which spans (−∞,∞).To yield detection probabilities, it was “squashed” through a likelihood function

(2) $$p_{\mathrm{yes}}\left(x_{*},x,y\right)=0.01+0.98Ф(f\left(x_{*},x,y\right))$$

with Ф denoting the Gaussian cumulative density function (CDF) and *p*_yes_ the probability of $x_{*}$ (a tone) being reported. Equation 2 produces values between 0.01 and 0.99, accounting for potential lapses in attention that lead to pressing the wrong button independent of $x_{*}$. The linear covariance was scaled so that the Gaussian CDF had a standard deviation of 3 dB, thus yielding a common shape for the psychometric functions.

Equation 1 requires approximate inference when used for classification. We did this using expectation propagation (Minka, 2001), with Laplace approximation (Williams & Barber, 1998) as a fall back when expectation propagation did not converge. We did not use variational inference (Bui et al., 2017; Hensman et al., 2013) because less than 100 data points were analyzed in each GP. The hyperparameters of a GP can be chosen based on the data already obtained by maximizing the marginal likelihood p(***y***|***x***,***θ***) with regard to the hyperparameters ***θ***. However, few data points are available at the start of a test, and optimization of all hyperparameters for the mean, covariance, and likelihood could lead to overfitting. Furthermore, early wrong responses can lead to wrong hyperparameter estimates at an early stage and thus instability in the BAL process. For this reason, only the hyperparameter of the mean function was optimized during the test; the other hyperparameters of the GP were fixed.

Modeling the response by a GP allows us to choose the parameters for the next trial efficiently. Intuitively one would place the level of the stimulus for the next trial close to threshold. However, the outputs of Equations 1 and 2 also give a variance, allowing us to choose regions where the current model is not “confident”. For the notched-noise test, there are two major sources for a lack of confidence: no or inadequate sampling of a certain frequency range and notch condition and inconsistent responses by the subject.

Ideally, the stimulus for the next trial should minimize the expected entropy in the model after the response for that trial has been made. Houlsby et al. (2011) showed that this gain in information can be expressed as the mutual information between the expected response y_*_ and the model *f* given the obtained data *D* (***x*** and ***y***) and the next data point $x_{*}$

(3) $$I\left(f,y_{*}|x_{*},D\right)=H\left(y_{*}|x_{*},D\right)-E_{f∼p\left(f|D\right)}[H\left(y_{*}|x_{*},D\right)]$$

In contrast to evaluating the expected entropy of the posterior directly, which requires evaluating one GP for each possible outcome and candidate data point, evaluating the expected entropy of the response (last term in Equation 3) requires only a single GP, using the data obtained already. Equation 3 provides an efficient way of looking one step ahead. Less myopic policies that look several steps ahead (e.g., Doire et al., 2017) may further speed up BAL procedures, but this is usually computationally intractable when using GPs.

### Information per Trial

The task that the subjects do, for example, indicating whether or not they have heard a tone or choosing one among several choices, has a direct impact on the information that can be obtained per trial, and thus the speed of a test. In a binary task such as responding “Yes” or “No,” the maximum information per trial is 1 bit. When additional catch trials are used to estimate any systematic response bias, the information that is gained about the threshold is reduced in proportion to the number of catch trials. For example, if 10% of all trials are catch trials, the maximum information per “average” trial is 0.9 bit.

**Figure 1.**
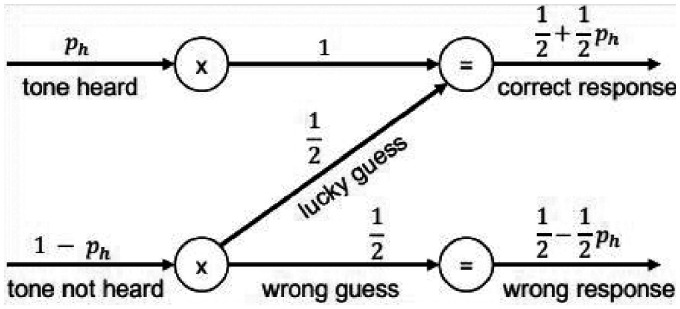
Model of the Response in a 2I-2AFC Task as a Binary Error Channel. The input is whether a tone is actually heard by the subject, the output the probabilities that she or he responded correctly.

Another popular task in psychophysics and specifically for experiments on auditory filters is the 2I-2AFC task. For a notched-noise test, a tone is presented in one of two intervals and the noise in both intervals. The subject has to indicate the interval in which the tone was presented. This procedure reduces the effects of the response criterion of the subject. However, correct responses may result from lucky guesses, which reduces the information gained per trial. The response can be modeled as a binary channel where one crossover probability is 0 (there is no wrong response when a tone is heard) and the other crossover probability is 0.5 when a tone is not heard (a lucky or correct guess). This response–channel model is shown in Figure 1. The information gained per trial without any prior knowledge is

(4) $$I=H_{b}\left(\frac{1}{2}+\frac{1}{2}p_{h}\right)-[\left(1-p_{h}\right)H_{b}\left(\frac{1}{2}\right)+p_{h}H_{b}\left(1\right)]$$

where *p_h_* is the probability that the tone is heard and *H_b_* is the binary entropy. The first term is the entropy of the output without prior knowledge. The second term is the entropy of the output when the input is known, which collapses to 1−*p_h_*. The first term increases with decreasing *p_h_,* but decreasing *p_h_* also leads to more being subtracted by the second term. *I* has a maximum (also known as the channel capacity) of 0.32 bits for *p_h_* = 0.60. Similarly, a 3I-3AFC task, which is sometimes used for notched-noise or similar experiments, yields maximum information of 0.47 bits at *p_h_* = 0.58 but requires one more sound presentation. This amount of information is still considerably less than for a Yes/No task, with up to 1 bit per trial, but the forced-choice methods have the advantage that responses are largely unaffected by the subject’s response criterion.

When estimating auditory-filter shapes, the response criterion effects in a Yes/No task can be accommodated by the “efficiency” parameter *K* (Patterson, 1976), leaving the shape parameters of interest by and large unaffected. This approach takes advantage of the time efficiency of a Yes/No task while at the same time not being prone to systematic biases in the slope parameters. Since we were mainly interested in the slope parameters and a 2AFC procedure gives considerably less information, we chose a Yes–No task for the BAL notched-noise test.

### Subjects

A total of 11 hearing-impaired subjects participated, 3 females and 8 males, aged 55 to 82 years (mean: 70 years). None reported any ear disease or trauma, except for S6 who reported having had a ruptured ear drum. They were paid to participate. They were tested using their better-hearing ear based on the mean audiometric threshold across 500 to 4000 Hz. Audiograms were obtained using the counting method of Schlittenlacher et al. (2018a). Audiograms are depicted by dashed lines in Figure 3, which is described later.

### Stimuli and Apparatus

The experiments took place in a double-walled sound-attenuating chamber. The stimuli were generated digitally with a sampling rate of 48000 Hz and a resolution of 24 bits, converted from digital to analog form by an M-Audio Delta 44 audio interface (Cumberland, RI), and attenuated by 15 dB with a manual attenuator. They were presented monaurally via a Sennheiser HDA200 headset (Wedemark, Germany).

The task was to detect a pure-tone signal in a notched-noise masker. The signal consisted of three pulses with a duration of 150 ms each and an interval of 100 ms between them. The duration of the noise was 850 ms. It started 100 ms before the first signal pulse and finished 100 ms after the last pulse. The signal pulses and the noise had 20-ms raised-cosine rise/fall times. The signal level (*L_s_*) was 15 dB SL and *f_s_* varied from 500 to 4000 Hz or the frequency at which the audiogram reached 40 dB HL for S1 to S6 or 50 dB HL for S7 to S11. The higher signal levels for S7 to S11 were allowed after estimating the loudness of the stimuli for S1 to S6, using the model of Moore and Glasberg (2004). Only 0.5% of the stimuli had a loudness level above 80 phon. For S7 to S11, 0.6% of the stimuli had a loudness level above 80 phon and none had a loudness level above 90 phon. The masker consisted of two noise bands, one centered below *f_s_* and one above, each with a bandwidth of 0.4*f_s_*. The frequency differences between *f_s_* and the upper edge of the lower noise band or the lower edge of the upper band were chosen to give five symmetric and four asymmetric notch conditions. These frequency differences, expressed as a proportion of *f_s_*, were (0|0), (0.1|0.1), (0.2|0.2), (0.3|0.3), (0.4|0.4), (0.1|0.3), (0.3|0.1), (0.2|0.4), and (0.4|0.2), chosen according to the recommendations of Stone et al. (1992). The level of the noise (*L_m_*) was an independent variable but was bounded so that at most 0.05% of the samples of the entire stimulus were clipped and the overall level was at most 95 dB SPL. *L_m_* was defined as the sound pressure level in a 1-Hz wide bin, that is, the spectrum level.

### Procedure

After the audiogram was obtained, the subjects did the notched-noise BAL test. Then, they repeated the notched-noise BAL test but using only the (0.2|0.2) notch, to check the consistency of the estimates. After this, notched-noise thresholds were determined using a 2-up/1-down procedure (Levitt, 1971) for the symmetric notches at *f_s_* = 1400 Hz, with the (0.2|0.2) notch in the second and last runs. The total test time was about 2 hr including breaks and all tests were conducted in one session.

### Notched-Noise BAL Test

There were three intervals in each trial, separated by 100 ms, containing in this order the signal only, the noise only, and the signal plus noise. This was done to allow the subject to know what to listen for, since the signal varied in frequency from trial to trial. The task was to indicate whether or not the signal was present in the third interval (Yes/No). Ten percent of the trials did not contain the signal in the third interval to give an estimate of false positives. While sounds were played, a blue rectangle appeared on the screen in the first and second intervals and a green rectangle in the third interval.

Before the BAL procedure commenced, *f_s_* and *L_m_* were chosen by simple rules for a few trials. The following procedure was repeated for each notch condition: (a) *f_s_* was 1000 Hz and *L_m_* was −20 dB SPL. *L_m_* was increased by 20 dB or decreased by 10 dB, depending on the response, and this was continued (but with the lower limit of *L_m_* set to −30 dB SPL) until a Yes and No response were obtained for *f_s_* = 1000 Hz; (b) *f_s_* was set to 2000 Hz and *L_m_* to the mean level used for the two previous trials; (c) *f_s_* was set to the highest frequency used with that subject and *L_m_* was set either 10 dB below or above the level used for *f_s_* = 2000 Hz, depending on the response for that frequency; thereafter, *L_m_* was decreased or increased by 10 dB until both a Yes and No response were obtained at this *f_s_*; and (d) *f_s_* was set to 500 Hz and a procedure similar to that for the highest frequency was used, except that *L_m_* was first set to the same value as used for *f_s_* = 2000 Hz. This typically required 10 trials or less per notch condition. The purpose of the initial grid was both to provide the GP with a rough initialization, which can be important when the actual threshold is not close to the prior mean, and to give the participants some practice with each notch condition, starting with a tone that was easy to detect.

After the initial grid was completed for each of the nine notch conditions, a GP was calculated for each notch condition. The hyperparameters of the GP were as described earlier: a Gaussian CDF likelihood function with lapse rates as described by Equation 2; a scalar constant mean across all *L_m_* and *f_s_* that was optimized before each trial so as to maximize the marginal likelihood of the data; a squared-exponential covariance in *f_s_* of 0.5 octaves; and a linear covariance in *L_m_* that was scaled by a factor of 3 to produce a standard deviation of 3 dB in the likelihood function. The inference function was expectation propagation (Minka, 2001), and Laplace (Williams & Barber, 1998) if the former did not converge. These settings are the same as those used by Schlittenlacher et al. (2018a). The GPs were implemented in Matlab using the GPML toolbox (Rasmussen & Nickisch, 2010).

The parameters for the next trial, namely, the notch condition, *f_s_*, and *L_m_*, were chosen to yield the highest mutual information about the threshold as a function of notch condition and *f_s_*. This was done as described earlier (Equation 3). The maximum was chosen out of nine GPs, one for each notch condition, instead of one (see also Houlsby et al., 2011). The minimum *L_m_* was set to −30 dB SPL and the maximum was set as described in the stimulus section. The minimum *f_s_* was set to 500 Hz and the maximum was between 2000 and 4000 Hz, as described in the stimulus section. Posterior distributions were calculated using the GP for all *L_m_* in this range with a step size of 1 dB, and for all frequencies with a step size of 0.1 octaves. Due to the distance-based covariance in frequency, the BAL procedure sampled more often at the edges of the frequency range than elsewhere because uncertainty about the response increased toward regions where no stimuli were presented (i.e., below the minimum *f_s_* or above the maximum *f_s_*). This effect was partially alleviated by including the edge frequencies in the initial grid. The procedure terminated after 594 trials (540 signal trials + 54 catch trials, an average of 60 per notch condition). The second run for the (0.2|0.2) notch terminated after 66 trials (60 signal trials and 6 catch trials). Subjects could see the progress of the experiment by a bar at the bottom of the screen.

### 2-Up/1-Down Tests

The staircase procedures described by Levitt (1971) are probably the most commonly used procedures in auditory tests. To compare our results with those obtained using one such procedure, thresholds were also estimated using a 2I-2AFC 2-up/1-down adaptive procedure for the symmetric notches, that is, (0|0), (0.1|0.1), (0.2|0.2), (0.3|0.3), and (0.4|0.4). The (0.2|0.2) notch condition was tested twice, as the second and last runs. The other notch conditions were run in random order. *L_s_* was 15 dB SL and *f_s_* was 1400 Hz. *L_m_* was changed in 5-dB steps until the second reversal, then in 3-dB steps until the fourth reversal, and in 1-dB steps thereafter. The procedure terminated after the 10th reversal. The average value of *L_m_* at the last four reversals was taken as the threshold.

## Results

**Figure 2.**
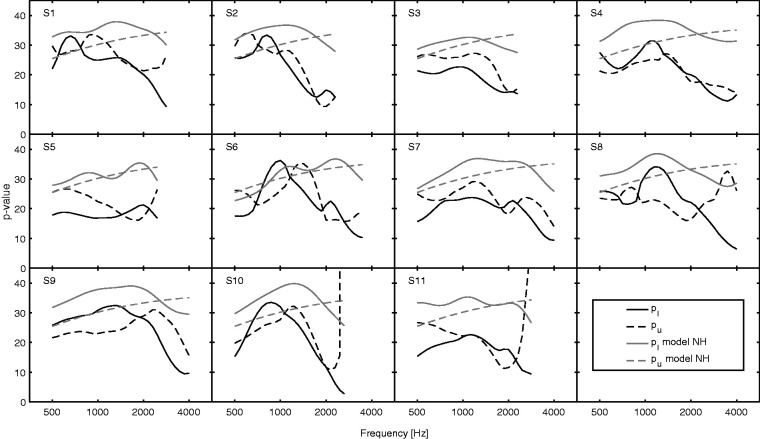
Black Lines Show Estimated Values of *p_l_* (Solid Lines) and *p_u_* (Dashed Lines). Gray lines show model predictions for normal-hearing subjects.

For the BAL notched-noise test, the value of *L_m_* at the 50% detection probability of the GP for each notch condition was taken as the threshold for that condition. This provided nine thresholds for each signal frequency, sampled in steps of 0.1 octaves. These were used to estimate auditory-filter shapes using a model with three parameters, *p_l_* and *p_u_*, which define the steepness of the lower and upper skirts, respectively, and *K*, which characterizes detection efficiency (Glasberg & Moore, 1990). This simple model does not allow for the flatter “tail” of the auditory filter, so the results for the (0.4|0.4) notch were not used in the analysis. The individual values of *p_l_* and *p_u_* are shown in Figure 2. Lower values indicate less sharp filters. For comparison, *p* values expected for normal hearing for the same signal levels (estimated using the model of Moore & Glasberg, 2004) are shown by light gray lines.

As expected, the *p_l_* and *p_u_* values (black lines) were generally smaller than expected for normal-hearing subjects, especially for the higher signal frequencies, for which the hearing losses were often greater. For S10 and S11, the value of *p_u_* increased markedly for the highest frequency tested, which is unrealistic. This reflects the fact that the upper slope of the auditory filter is not well defined using the notched-noise method when the lower slope is very shallow (Glasberg & Moore, 1990).

**Figure 3.**
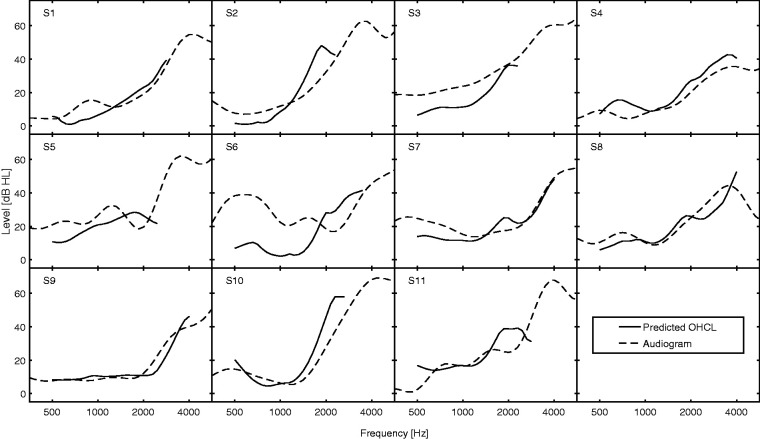
Solid Lines Show OHCL Values Derived From *p_l_* and *p_u_* Using the Model of Moore and Glasberg (2004). Dashed lines show the audiometric thresholds. OHCL = outer hair cell dysfunction.

The *p_l_* and *p_u_* values can be related to the amount of hearing loss due to outer hair cell dysfunction (OHCL), using the model of Moore and Glasberg (2004); smaller values of *p_l_* and *p_u_* indicate greater OHCL. Figure 3 shows these relations. For a typical cochlear hearing loss, OHCL is about 90% of the audiometric threshold for hearing losses up to about 55 dB. Consistent with this, the estimated values of OHCL were usually close to the audiometric thresholds, except for S6, who probably had a conductive component to her hearing loss.

**Figure 4.**
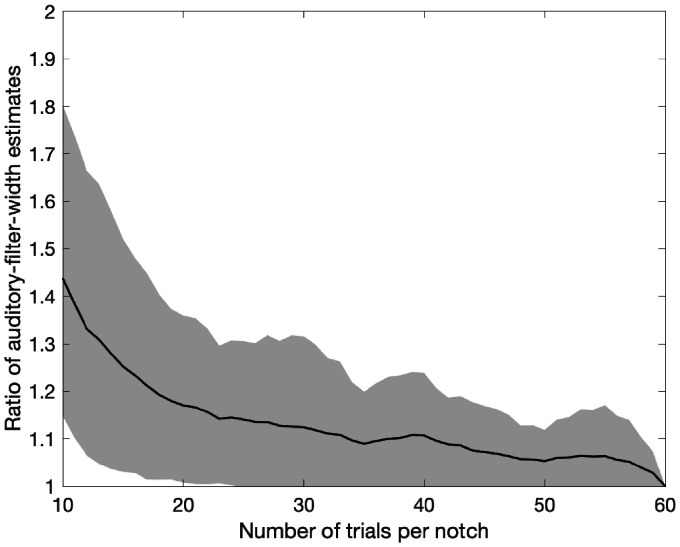
Ratio Between Estimated Auditory-Filter Width After *x* Trials Per Notch and the Final Estimate, Plotted as a Function of *x*. The inverse was taken if the ratio was smaller than 1. The solid line shows the geometric mean across subjects and the gray area shows the geometric standard deviation.

To use the test in a clinical application, it would be desirable to terminate it as soon as sufficient accuracy is reached. The experiment with an average of 60 trials per notch condition took 48 to 61 min. The estimated auditory-filter width was calculated after each trial and divided by the final estimate. The inverse was taken if the ratio was smaller than 1. Figure 4 shows the geometric mean ratio across subjects. The ratio drops below 1.12, representing a small error and corresponding to a discrepancy in OHCL of about 5 dB, after 30 trials per notch condition. For comparison, test–retest differences in an audiogram are also about 5 dB (Margolis et al., 2010). A total of 30 trials for 8 notches could be obtained in about 20 to 30 min.

**Figure 5.**
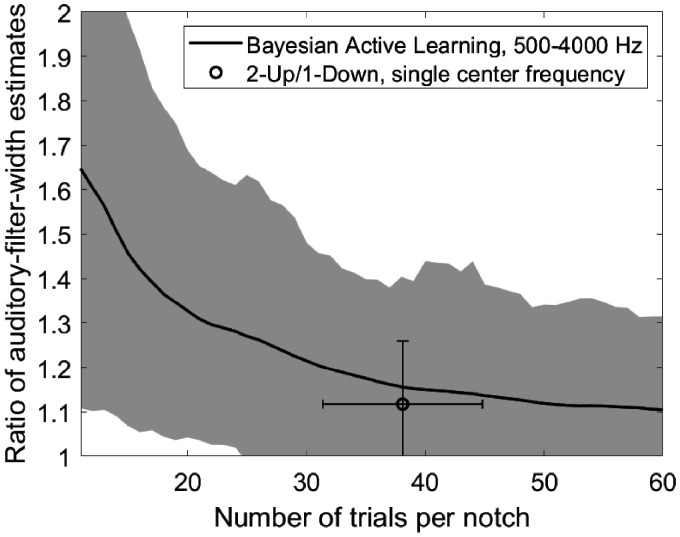
Ratio Between Estimated Auditory-Filter Width After *x* Trials Per Notch Condition and the Ground Truth for Simulated Responses. The inverse was taken if the ratio was smaller than 1. Responses were simulated taking the actual final thresholds and incorporating a lapse rate of 1% and a Gaussian CDF psychometric function with a standard deviation of 3 dB. The solid line shows averages across center frequency, subjects, and 10 simulated runs. The circle shows results of a simulation for a 2-up/1-down procedure (Levitt, 1971) that yields the auditory-filter shape for a single center frequency. The simulation parameters, with runs terminated after 10 reversals, and the choice of notch conditions were as proposed by Stone et al. (1992).

Figure 4 compares the filter-width estimates after a given number of trials to the estimates after the last trial, that is, not to an independent ground truth. Simulations were conducted to overcome this limitation. The thresholds estimated after the last trial of the actual experiment were taken as the ground truth for the simulation. Responses were simulated with a lapse rate of 1% and a Gaussian CDF with a standard deviation of 3 dB for the psychometric function. Ten runs were simulated for each subject. As for Figure 4, auditory-filter shapes were calculated after each trial, and the ratio of filter widths to those for the ground truth is shown in Figure 5. After 30 trials, the ratio is 1.20, which corresponds to a discrepancy in OHCL of about 8 dB.

The test duration may be divided into four parts: stimulus presentation, response time, intertrial interval, and breaks. A total test duration of 48 to 61 min yields an average of 5.3 to 6.8 s per trial. Response times were measured as the interval between the end of the third stimulus and the mouse click. There was no button for a break but subjects were instructed to move the mouse over the response button but not to click it in this case, so breaks could be detected as long response times. There were 1.2 trials per subject with response times longer than 60 s, and 5.4 trials per subject with response times between 5 and 60 s. The mean of all response times that were shorter than 5 s was 1.0 s (standard deviation: 0.6 s). Stimulus presentation took 2.75 s. Intertrial intervals were not measured and were mainly determined by the time needed to calculate the GP on a single processor unit. They lasted up to about 4 s for the final trials.

Despite not being forced to take a break during the test, the subjects showed few lapses of attention. They responded “Yes” to 0 to 2 of the 54 catch trials (mean: 0.64 of 54; 1.2%). The steepness of the psychometric function is represented by the standard deviation of the Gaussian CDF that is used for the likelihood function. To estimate this parameter, it was optimized to maximize the probability of the data (in the same way as the hyperparameter for the mean was optimized) for each of the 99 (11 Subjects × 9 Notch Conditions) GPs after all trials were completed, and then averaged across notch conditions for each subject. The mean of this measure was 2.4 dB, with a range from 1.4 dB to 3.5 dB. Thus, both the actual lapse rate and the steepness of the psychometric function were close to the prior assumption that was used in the experiment, 1% and 3 dB, respectively.

**Figure 6.**
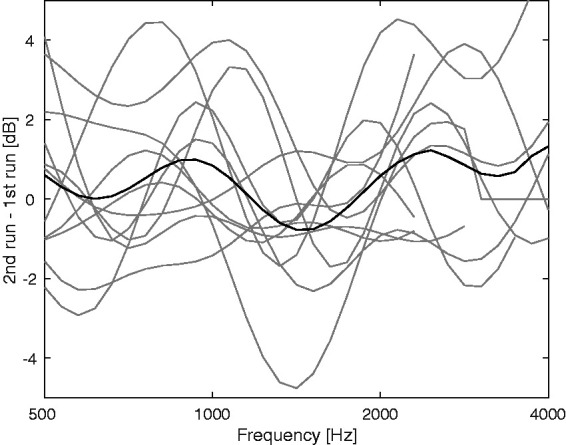
Difference Between the Threshold for the Second BAL Test for the (0.2|0.2) Notch only and the Threshold for That Notch Condition Obtained in the Main Test. The black and gray lines show the mean and individual results, respectively.

The BAL procedure was rerun using the (0.2|0.2) notch width to assess consistency and repeatability. The differences between main test and retest are shown in Figure 6. The average difference was 0.4 dB and the root-mean square difference (RMSD) was 1.8 dB. The slightly higher mean noise level at threshold for the second runs may indicate a small learning effect.

**Figure 7.**
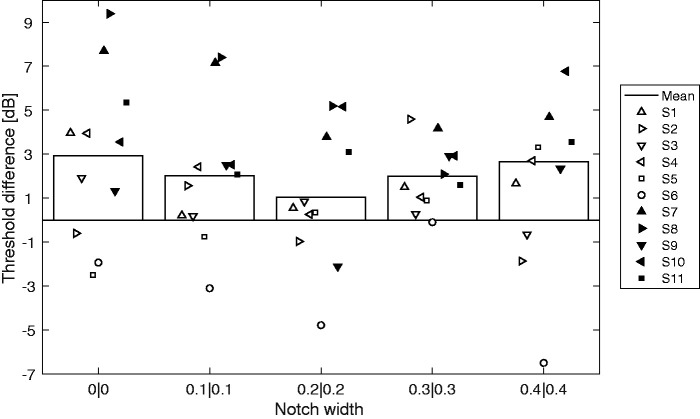
Difference Between the Thresholds at 1.4 kHz Obtained Using the 2-Up/1-Down Procedure and the BAL Method for the Five Symmetric Notches. Bars show the mean across subjects and symbols show individual results.

To compare the BAL method with a conventional procedure, thresholds for the five symmetric notch conditions were estimated at 1.4 kHz using a 2I-2AFC 2-up/1-down procedure. The differences between thresholds obtained with this procedure and with the BAL method are shown in Figure 7. The overall difference was 2.1 dB and the RMSD was 4.0 dB. A certain systematic difference may be expected because the response criterion affects thresholds in the Yes/No procedure. However, the difference did not vary significantly across notch conditions, as confirmed by a within-subjects analysis of variance, *F*(4,40) = 1.25, *p *=* *.31, η_p_^2^ = 0.11, suggesting that the threshold differences are systematic and would mainly lead to a difference in parameter *K*, but not in the filter slopes. The mean difference between the first and second runs for the (0.2|0.2) notch with the 2-up/1-down procedure was 0.2 dB and the RMSD was 1.2 dB.

Simulations were also done for a 2I-2AFC 2-up/1-down method and are shown by the circle in Figure 5. For this simulation, the thresholds of all subjects and all frequencies for the (0|0), (0.2|0.2), (0.4|0.4), (0.2|0.4), and (0.4|0.2) notches that were obtained in the behavioral BAL method were taken as ground truth. Responses were simulated in the same way as for the simulated BAL method, with a lapse rate of 1% and a Gaussian CDF with a standard deviation of 3 dB for the psychometric function. A simulated run terminated after 10 reversals and the mean of the masker levels at the last 4 reversals was taken as the threshold. As suggested by Stone et al. (1992), thresholds were averaged across two runs before auditory-filter shapes were calculated. The circle in Figure 5 shows the average ratio between the auditory-filter width obtained in the simulation and the ground-truth auditory-filter width. Error bars show the standard deviations in number of trials needed for one run with one notch condition (horizontal) and the geometric standard deviation of the ratio of auditory-filter widths. The 2-up/1-down method is slightly more accurate than the BAL method after an equal number of trials. However, the 2-up/1-down method estimates only a single auditory filter for one center frequency while the BAL method estimates auditory filters across a wide range of center frequencies.

## Discussion

The proposed BAL notched-noise method proved to be consistent; thresholds for the (0.2|0.2) notch were similar when estimated in isolation or as part of the main procedure including all notch conditions. Furthermore, differences between the BAL method and the 2-up/1-down procedure were similar across notch conditions, with an effect size of notch condition of only η_p_^2^ = 0.11. Systematic differences in threshold across conditions mainly affect the parameter *K*, reflecting the combined effects of detection efficiency and response criterion.

The focus of the BAL method was on the estimation of thresholds that could be used for calculating auditory-filter shapes. The method was not designed to make use of knowledge about the parameters of the underlying auditory filters (in contrast, e.g., to Shen and Richards, 2013, and the dead-region test of Schlittenlacher et al. 2018b). Knowledge of the model parameters could be used to select informative notch configurations and hence might be somewhat faster. However, the present approach allowed comparisons to traditional tests with regard to systematic biases, none of which were found to affect auditory-filter shapes.

Instead of using nine independent two-dimensional GPs, one could use a single three-dimensional GP, exploiting covariance between thresholds for the different notch conditions and possibly making the test even faster. However, low-dimensional GPs have the advantage of being computationally less expensive, an important aspect given the extensive computation that is required between trials. Furthermore, only one of the nine GPs needed to be updated after each trial. The current test could be speeded up a little by using optimized code and more than one central processing unit (CPU), since the intertrial interval was longer than the interval that is typically used in experiments (200–1000 ms) due to the time required to compute the GP.

The results could be used to estimate the subjects’ psychometric functions by optimizing the GPs with regard to the corresponding hyperparameter during the experiment. However, the present test did not sample informatively with regard to that aim. If this was desired in a BAL test, the policy for choosing the next trial would need to incorporate both the threshold and variance of the psychometric function (Brand & Kollmeier, 2002; Song et al., 2017). The estimated steepness of the psychometric function after the completion of the experiment (standard deviation of a Gaussian CDF) of 2.4 dB on average was close to the value of 3 dB assumed for our test but was somewhat larger than the value of 1.5 dB found by Schlittenlacher et al. (2018a) for absolute thresholds. The psychometric function for the detection of a tone in noise may be more shallow than that for the detection of a tone in quiet.

Both the comparison of the auditory-filter width to the result after the last trial (Figure 4) and comparison to a ground truth in simulations (Figure 5) showed that the accuracy was reasonably good after about 30 trials, with no marked improvement thereafter. This number of trials can be done in less than 30 min, yielding auditory-filter shape estimates across three octaves.

The estimates of auditory- filter shape might be useful in determining the frequency- and level-dependent gains to be used when fitting multichannel compression hearing aids. Currently, methods for prescribing these gains are primarily based on audiometric thresholds (Keidser et al., 2011; Moore et al., 2010; Scollie et al., 2005). However, the methods were developed using auditory models for impaired hearing, such as that of Moore and Glasberg (2004), and one goal of the methods is to minimize masking across different frequency regions. Specifically, the frequency- and level-dependent gains are intended to avoid any given frequency band from having a strong masking effect on adjacent bands (Fletcher, 1953). The models used to develop the prescription methods were based on “default” or average parameters for inner and outer hair cell loss. However, fittings might be more effective if the parameters characterizing an individual’s hearing were known. Auditory-filter measurements represent one step toward this. For example, if the auditory filters have unusually shallow low-frequency slopes, it might be advantageous to make the gain increase relatively strongly with increasing frequency to reduce the upward spread of masking from low frequencies to higher frequencies.

## Conclusions

BAL methods have the potential to introduce tests into clinical practice that previously took too much time. In addition, they increase the information provided, since they are not limited to a grid. The BAL notched-noise test described here has been shown to be reliable, valid, and rapid, making it feasible for clinical use and also useful for scientific research, allowing more information to be collected in a given amount of experimental time. Compared with other psychophysical methods, the present BAL method has the main advantage that it allows the determination of auditory-filter shapes over a range of frequencies rather than only at a few discrete center frequencies.

The analysis method used here circumvented the effect of systematic biases that can occur in yes–no tasks by using an auditory-filter model to interpret the results rather than by directly interpreting the obtained thresholds.

Auditory-filter shape estimates over a range of frequencies may be useful for characterization of an individual’s hearing and for more personalized initial fitting of a hearing aid. Together with other BAL tests for the audiogram, dead regions, or fine-tuning an initial fitting (see the introduction of this article), this provides potential tools for personalized precision medicine.
